# Sex-specific-evaluation of metabolic syndrome prevalence in Algeria: insights from the 2016–2017 non-communicable diseases risk factors survey

**DOI:** 10.1038/s41598-023-45625-y

**Published:** 2023-11-02

**Authors:** Calypse Ngwasiri, Mikaila Kinoré, Sekou Samadoulougou, Fati Kirakoya-Samadoulougou

**Affiliations:** 1 Clinical Research Education Networking and Consultancy (CRENC), RFMR+QFH, Yaoundé, Centre Region Cameroon; 2 Centre de Recherche en Epidémiologie, Biostatistique et Recherche Clinique, Ecole de Santé Publique, Université libre de Bruxelles, Brussels, Belgium; 3 Département de Biochimie et Microbiologie, UFR-SVT, Université Joseph Ki-Zerbo, Ouagadougou, Burkina Faso; 4 Centre for Research on Planning and Development, Université Laval, Quebec, QC G1V 0A6 Canada

**Keywords:** Public health, Lifestyle modification, Preventive medicine

## Abstract

Metabolic syndrome (MetS) is a core driver of cardiovascular diseases (CVD); however, to date, gender differences in MetS prevalence and its components have not been assessed in the Algerian adult general population. This study aimed to determine the gender differences in MetS prevalence and its components, in the general population of Algeria. Secondary analysis was performed on data from the Algerian 2016–2017 non-communicable disease risk factor survey. MetS was determined according to the harmonized Joint Interim Statement criteria. A Poisson regression model based on Generalised Estimating Equations was used to estimate the adjusted prevalence ratios (aPR) for the sex-specific factors associated with MetS. Overall, the prevalence of MetS was 34.0% (95% CI 32.4–35.6). MetS prevalence in women and men was 39.1% (95% CI 37.0–41.3) and 29.1% (95% CI 27.2–31.2), respectively. The most frequent triad was the clustering of abdominal obesity with low HDL-cholesterol and high blood pressure among women (8.9%; 95% CI [8.0–10.0]) and low HDL-cholesterol with high blood pressure and hyperglycaemia among men (5.2%; 95% CI [4.3–6.3]). Increasing age (aPR 3.21 [2.35–4.39] in men and aPR 3.47 [2.86–4.22] in women), cohabitation (aPR 1.14 [1.05–1.24]), women residing in urban areas (aPR 1.13 [1.01–1.26]), men with higher educational levels (aPR 1.39 [1.14–1.70]), and men with insufficient physical activity (aPR 1.16 [1.05–1.30]) were associated with higher risk of MetS. In this population-based study, one in three Algerian adults had MetS, and key components including abdominal obesity, low HDL-cholesterol, and high blood pressure, are very common, especially in women. Reinforcing interventions for weight management targeting married women living in urban areas and improving sufficient physical activity in men with higher socioeconomic status could provide maximal health gains and stem the CVD epidemic in Algeria.

## Introduction

Metabolic syndrome (MetS) refers to the clustering of interconnected disorders, including hyperglycemia, central obesity, high blood pressure, low levels of high-density lipoprotein cholesterol (HDL-cholesterol), and elevated triglycerides^1^. MetS is a key driver of the global cardiovascular epidemic and significantly increases the risk of morbidity and mortality from cardiovascular disease (CVD)^2,3^ and type 2 diabetes (T2D)^4^. MetS is believed to arise from an interplay between environmental and genetic factors that lead to excess adiposity and subsequently to other metabolic derangements^5^. Worldwide, MetS prevalence ranges from 12.5% to 31.4% in the general adult population^6^ and is a public health concern given that metabolic risks contributed the most attributable deaths and disability-adjusted life years in 2019^7,8^.

Variations in MetS prevalence are due to the heterogeneity in the various diagnostic criteria, which are mainly glucocentric or obesity-centric (World Health Organization [WHO] and International Diabetes Federation [IDF] criteria) or assembled around CVD prediction (National Cholesterol Education Program Adult Treatment III [NCEP ATP III] criteria). Regardless of the criteria used, the presence of at least three metabolic components is diagnostic for MetS^1^. MetS prevalence is highest in the Eastern Mediterranean and Americas, and increases with a country’s income level^6^. In Sub-Saharan Africa (SSA), MetS is common (pooled prevalence of 11.1% to 23.9%), and the most at-risk groups include urban populations, populations residing in Southern Africa, and women^9^.

Data on MetS have not yet been systematically assessed in North Africa; however, the available evidence suggests that MetS prevalence might be even higher than that reported in SSA. Two nationwide studies from Tunisia and Morocco estimated MetS prevalence in the general adult population as 30%^10^ and 40%^11^, respectively. Single-centre studies in Libya revealed that more than 80% of patients with T2D had MetS^12^ and that MetS was also highly prevalent in women without diabetes^13^. In Algeria, published primary studies on MetS have been fragmented and have focused on specific population groups. A survey conducted on residents in a semi-rural area in northwest Algeria revealed that MetS prevalence was 17.4% (NCEP-ATP III) and 25.7% (IDF) in the adult population in 2009^14^. More recently, studies on MetS have focused on patients with rheumatoid arthritis^15^, adults in a suburban area (Algiers)^16^, and insured city residents in an urban area in North Algeria(Ora)^17^. Across these studies, MetS was more prevalent in women, and the most common components were low HDL cholesterol and abdominal obesity in women and high blood pressure in men.

Data on the nationwide MetS prevalence in the adult Algerian population as well as information on the gender differences in MetS components are lacking. In this study, we aimed to determine the prevalence rates of MetS and its individual components according to the harmonised criteria and cutoffs in the Algerian general population and to investigate gender differences and factors associated with MetS among men and women in Algeria.

## Materials and methods

### Data source and study population

This study used data from the Algerian STEPwise approach to Non-Communicable Disease (NCDs) risk factor surveillance (STEPS) survey, implemented by the Ministry of Health with the support of the WHO. STEPS is a WHO-recommended household-based survey for countries to obtain core data on the established risk factors that determine the major burden of NCDs. Two STEPS surveys have been conducted in Algeria (2003 and 2016–17), and the study protocols were approved by the National Council for the Ethics of Health Sciences review board. These cross-sectional surveys were carried out in compliance with the guidelines and regulations of the Helsinki Declaration, and written informed consent was obtained from all participants. Detailed study protocols, including the data collection methods have been published elsewhere^18,19^.

Briefly, the STEPS covers three different steps of risk factor assessment including home interviews, physical measurements, and biochemical measurements. For each survey, a multi-stage cluster sampling design was used to obtain representative data. The first stage of the sampling process involved the selection of primary sampling units (districts) followed by the selection of specific enumeration areas within these districts that contain a cluster of households. In the third stage, specific households within these enumeration areas were identified, and one individual within the age range of the survey was selected per household. Analysis weights were used to adjust the data so that it is representative of the target population. These weights were calculated by taking the inverse of the probability of selection of each participant and adjusted for differences in the age-sex composition of the sample population as compared to the target population. Different weight variables were used for each step of the survey to allow for differences in weight calculation due to participant refusal or drop out^19^.

**Figure 1:**
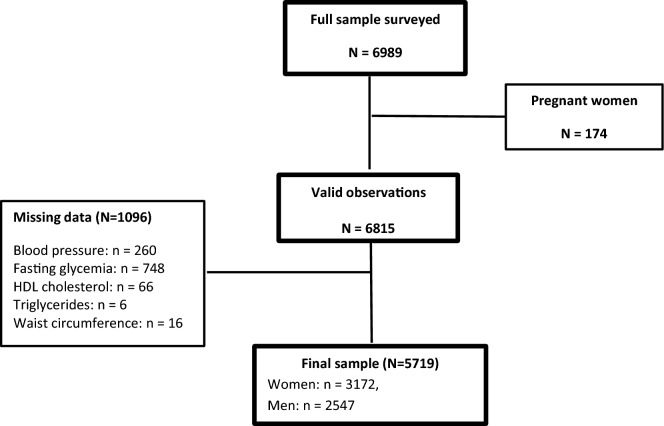
Participant flow chart.

For the present study, secondary analysis was conducted on data obtained from the second STEPS survey (2016–2017) and included adults aged 18–69 years who had undergone home interviews, physical measurements, and biochemical measurements. Pregnant women and those with established CVD were excluded from the study. A total of 6989 adults participated in the survey, and the overall response rate was 93.8%. Overall, 5719 adults were eligible and had no missing data for any of the five MetS components (Fig. 1).

### Data collection and measures

In this survey, all 3 levels of risk factor assessment were performed. Sociodemographic data (including age, sex, level of education, work status, and place of residence) and behavioural information (tobacco use, healthy diet, and physical activity) were collected in step 1. In the second step, weight, height, waist circumference (WC), and blood pressure measurements were collected, whereas biochemical measurements to assess blood cholesterol and sugar levels were done in step 3. Details of the data collection procedure have been previously described^18^. Briefly, after at least 10 min of rest, the blood pressure of each participant was measured three times in a seated position, 5 min apart, and the mean value of the second and third readings was considered as the participant’s blood pressure. A portable constant tape (precision of 0.1 cm) was used to measure height and WC in a standing position whilst weight was measured using a digital scale and recorded to the nearest 0.1 kg (kg). Body mass index (BMI) was calculated as the weight in kg divided by the height in square meters. Finally, fasting venous blood samples were obtained from each participant after an overnight fast to assess fasting plasma glucose (FPG) and blood lipids via the enzymatic colorimetric method.

### Definition of MetS and variables

The harmonized MetS definition from the Joint Interim Statement^5^ was used which constitutes the presence of any 3 of 5 risk factors including: central obesity, hypertension or antihypertensive drug treatment, elevated FPG or drug treatment for elevated glucose, elevated serum triglyceride or drug treatment for elevated triglyceride, and low HDL-cholesterol or drug treatment for reduced HDL-cholesterol (Table 1). Waist circumference for central obesity measurement was defined according to the Middle Eastern and Mediterranean population thresholds for men and women, whilst physical activity levels and daily fruit and vegetable consumption were assessed using the WHO guidelines^20^ (Table 1).

**Table 1:** Definition of MetS components and behavioural factors.

Variable	Measure	Categorical cut points
Abdominal obesity	Elevated waist circumference	Yes: ≥ 80 cm (women); ≥ 94 cm (Men)
		No: ˂ 80 cm (women); ˂ 94 cm (Men)
High blood pressure	Elevated blood pressure or use of antihypertensive medication	Yes: SBP ≥ 130 mmHg and or DBP ≥ 85 mmHg
		No: SBP < 130 mmHg and or DBP < 85 mmHg
Hypertriglyceridemia	Elevated triglycerides or drug treatment for elevated triglycerides	Yes: ≥ 150 mg/dl;
		No: ˂ 150 mg/dl
Low HDL cholesterol	Reduced HDL-cholesterol or drug treatment for reduced HDL-cholesterol	Yes: ˂ 50 mg/dl (Women) or ˂ 40 mg/dl (Men)
		No: ≥ 50 mg/dl (Women) or ≥ 40 mg/dl (Men)
Hyperglycaemia	Elevated fasting glucose or drug treatment for elevated glucose	Yes: ≥ 100 mg/dL and/or treatment
		No: ˂ 100 mg/dL
Physical activity	Sufficient: ≥ 1500 metabolic equivalent of task (MET)-min/week	Yes: ≥ 1500 MET min/week
	Insufficient: < 1500 MET-min/week	No: < 1500 MET-min/week
Fruit and vegetable consumption	Sufficient: consume at least 5 servings of fruit and vegetables per day	Sufficient: ≥ 5 servings of fruit and vegetable /day
	Insufficient: consume less than 5 servings of fruit and vegetables per day	Insufficient: < 5 servings of fruit and vegetable /day
Smoking	Non-smoker: never smoked nor used smokeless tobacco	Non-smoker, past smoker, current smoker
	Past smoker: smoked in the past	
	Current smoker: current smoker/ used smokeless tobacco in the past 30 days	

### Covariates

The independent variables included in the analysis were age, sex, education level, living status, occupation, and place of residence. Age was categorised as (18–29 [reference], 30–44, 45–59, and 60–70 years), level of education (less than primary, primary, secondary, and university [reference]), living status (living alone, cohabiting [reference]), employment (unemployed [reference] and employed), and residence (urban [reference] and rural).

### Data processing and analysis

Statistical analyses were performed using STATA version 17.0. For descriptive statistics, categorical variables were summarised as frequencies and proportions, and the estimates accounted for the complex nature of the survey design using the different sampling weights.

To evaluate the association between MetS (as an outcome, using the harmonized criteria) and the individual sociodemographic (age, level of education, living status, occupation, and place of residence) and behavioural factors (smoking, physical activity, and fruit and vegetable consumption) crude and adjusted prevalence ratios (aPR) were estimated. First, univariable Poisson regression models based on Generalized Estimating Equations with robust standard errors and clustering by primary sampling unit to estimate crude prevalence ratios (cPR) and its 95% confidence intervals (CIs) were used^21,22^.

Multivariable analysis was performed and adjusted for individual covariates, and the adjusted prevalence ratio (aPR), 95% CI, and P-values were generated to determine the sex-specific predictors of MetS and its components in Algeria. A statistical significance was set at P < 0.05.

Reporting was performed according to Strengthening the Reporting of Observational Studies in Epidemiology (STROBE) guidelines for cross-sectional studies.

## Results

Overall, 6989 participants were included, of whom three out of four adults met the eligibility criteria. Therefore, 2547 men and 3172 women were included in the final analysis (Fig. 1). Table 2 summarises the baseline characteristics of the study population according to sex. The mean age of the study population was 38.2 ± 12.4 years, and more than two-thirds of the participants cohabited. Most people were unemployed, lived in urban areas, and had insufficient levels of daily fruit and vegetable consumption (Table 2).

**Table 2:** Baseline characteristics of the study population.

Participant characteristics	All (N = 5719) frequency (%)	Women (N = 3172) frequency (%)	Men (N = 2547) frequency (%)
All participants	5719 (100)	3172 (48.7)	2547 (51.3)
Age group, years			
18–29	1072 (28.4)	612(28.1)	460 (28.6)
30–44	2349 (40.6)	1316 (40.2)	1033 (41.0)
45–59	1533 (21.4)	806 (21.9)	727 (21.0)
60–70	765 (09.6)	438 (09.8)	327(09.4)
Living status			
Living alone	689 (37.0)	1076 (36.9)	1765 (37.0)
Cohabiting	3948 (63.0)	2092 (63.1)	1856 (63.0)
Completed level of education			
Less primary school	1885 (27.9)	1238 (34.2)	647 (21.9)
Primary school	1359 (25.4)	608 (19.7)	751 (30.8)
Secondary school	1708 (32.2)	856 (29.2)	852 (35.0)
University studies	752 (14.5)	461 (16.9)	291 (12.3)
Occupation			
Employee	2270 (43.0)	503 (16.5)	1767 (68.2)
Unemployed	3439 (57.0)	2663 (83.5)	776 (31.8)
Residence			
Urban	3823 (66.5)	2138 (66.6)	1685 (66.3)
Rural	1896 (33.5)	1034 (33.4)	862 (33.7)
Smoking			
Non-smoker	4118 (69.1)	3150 (99.4)	968 (40.2)
Past smoker	822 (14.6)	9 (0.3)	813 (28.3)
Current smoker	775 (16.3)	11 (0.3)	764 (31.5)
Alcohol			
Non-user	5250 (90.9)	3159 (99.7)	2091 (82.6)
User	464 (9.1)	10 (0.3)	454 (17.4)
Fruits and vegetables			
Sufficient	1344 (23.6)	734 (23.1)	610 (24.0)
Insufficient	4375 (76.4)	2438 (76.9)	1937 (76.0)
Sufficient physical activity			
Yes	3339 (61.2)	1494 (47.4)	1845 (74.4)
No	2375 (38.8)	1675 (52.6)	700 (25.6)

### Metabolic syndrome prevalence, MetS components, and co-occurrence

**Figure 2:**
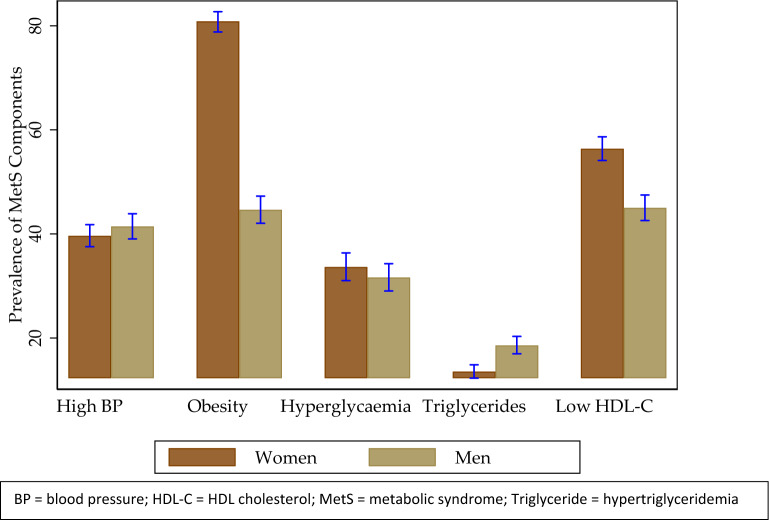
Prevalence of MetS components in the Algerian general adult population.

In the Algerian general adult population, the overall MetS prevalence in individuals aged 18 years and over in 2017 was 34.0% (95% CI 32.4–35.6) according to the harmonized definition. In addition, MetS prevalence was higher in women (39.1% [95% CI 37.0–41.3]) compared to men (29.1% [95% CI 27.2–31.2]). Figure 2 summarises and compares the frequencies of MetS components in men and women. In women, the most frequent MetS components were abdominal obesity (80.8%; 95% CI 78.8–82.7), low HDL cholesterol (56.4%; 95% CI 54.1–58.7) and high blood pressure (39.7%; 95% CI 37.6–41.8), whereas in men, the most frequent components were low HDL cholesterol (45.0% 95% CI 42.6–47.5), abdominal obesity (44.6%; 95% CI 42.1–47.3) and high blood pressure (41.4%; 95% CI 39.1–43.9). Regarding the other MetS components, the prevalence of hyperglycaemia and hypertriglyceridemia was 33.6% (95% CI 31.0–36.4) and 13.5% (95% CI 12.3–14.9), respectively, in women, and 31.6% (95% CI 29.1–34.3) and 18.6% (95% CI 17.0–20.3), respectively, in men (Fig. 2).

Table 3 shows the sex-specific prevalence of co-occurrence of MetS components. Overall, the most common triad was low HDL cholesterol, high blood pressure, and hyperglycaemia (6.7%; 95% CI 5.9–7.6), followed by abdominal obesity, low HDL cholesterol, and high blood pressure (6.6%; 95% CI 6.0–7.3), regardless of sex. None of the participants had more than four MetS components.

**Table 3:** Prevalence of the co-occurrence of MetS components by sex in the Algerian adult population.

Participant characteristics	N	Overall(N = 5719)	Women (N = 3172)	Men (n = 2547)
		Prevalence [95%CI]	Prevalence [95%CI]	Prevalence [95%CI]
Abdominal obesity + Low HDL + High BP	432	6.6 [6.0–7.3]	8.9 [8.0–10.0]	4.4 [3.6–5.3]
Abdominal obesity + Low HDL + Hyperglycaemia	234	3.8 [3.2–4.3]	5.3 [4.5–6.3]	2.2 [1.7–2.9]
Abdominal obesity + Low HDL + Hypertriglyceridemia	106	1.9 [1.5–2.3]	2.0 [1.5–2.6]	1.7 [1.3–2.3]
Abdominal obesity + High BP + Hyperglycaemia	283	4.1[3.6–4.6]	4.5 [3.9–5.3]	3.6 [3.0–4.4]
Abdominal obesity + High BP + Hypertriglyceridemia	91	1.4 [1.1–1.8]	1.2 [0.8–1.6]	1.7 [1.2–2.3]
Abdominal obesity + Hyperglycaemia + Hypertriglyceridemia	52	0.9 [0.7–1.2]	0.9 [0.6–1.2]	0.9 [0.6–1.3]
Low HDL + High BP + Hyperglycaemia	457	6.7 [5.9–7.6]	8.3 [7.2–9.5]	5.2 [4.3–6.3]
Low HDL + High BP + Hypertriglyceridemia	149	2.4 [2.0–2.8]	1.9 [1.5–2.5]	2.8 [2.2–3.5]
Low HDL + Hyperglycaemia + Hypertriglyceridemia	100	1.7 [1.4–2.1]	1.6 [1.2–2.1]	1.9 [1.4–2.6]
High BP + Hyperglycaemia + Hypertriglyceridemia	313	4.6 [4.0–5.3]	4.5 [3.8–5.4]	4.7 [3.9–5.6]

### Factors associated with MetS in Algeria

Tables 4 presents the sex-specific determinants of MetS in Algeria. In both men and women, after adjusting for all other variables, increasing age was significantly associated with MetS. Compared with younger adults (age 18–29 years), MetS prevalence was three times higher among individuals aged 60–70 years (aPR 3.21 [2.35–4.39]; P <  0.001 in men, and aPR 3.47 [2.86–4.22]; P < 0.001 in women, respectively). Living status was also significantly associated with MetS regardless of sex, whereas being married or cohabiting increased the risk of MetS by approximately 33% (aPR 1.33 [1.07–1.63]; P = 0.009). Women who resided in urban areas had a 1.13-times (aPR 1.13 [1.02–1.26]; P = 0.023) higher MetS prevalence than those living in rural areas, and men who had a higher level of education had a MetS prevalence that was approximately 1.3-times higher (aPR 1.34 [1.11–1.61]; P = 0.015) than those without formal education. Insufficient physical activity was the only behavioural factor associated with MetS in this population, and this association was observed only in men. Men who did not engage in sufficient levels of physical activity had about a 1.2-times higher (aPR1.16 [1.05–1.30]; P = 0.005) MetS prevalence than their physically active counterparts (Table 4).

**Table 4:** Sociodemographic and behavioural characteristics associated with MetS among women and men in Algeria.

Characteristics	N	MetS prevalence	cPR (95%CI)	P	aPR (95%CI)	P	N	MetS prevalence	cPR (95%CI)	P	aPR (95%CI)	P
	Women						Men					
All participants	3172	39.1 [37.0–41.3]					2547	29.1 [27.2–31.2]				
Age group, years												
18–29	612	17.8 [14.9–21.2]	1	** < 0.001**	1	** < 0.001**	460	12.0 [9.3–15.4]	1	** < 0.001**	1	** < 0.001**
30–44	1316	35.3 [32.5–38.3]	2.00 [1.67–2.39]		1.88 [1.57–2.26]		1033	28.3 [25.4–31.5]	2.31 [1.77–3.01]		1.91 [1.42–2.56]	
45–59	806	58.7 [54.9–62.3]	3.26 [2.72–3.89]		2.93 [2.43–3.55]		727	44.3 [40.7–48.0]	3.58 [2.77–4.63]		2.78 [2.06–3.76]	
60–70	438	72.0 [67.1–76.5]	3.93 [3.28–4.70]		3.47 [2.86–4.22]		327	51.0 [45.9–56.1]	4.10 [3.17–5.31]		3.21 [2.35–4.39]	
Living status												
Living alone	1076	30.5 [27.6–33.7]	1	** < 0.001**	1	**0.003**	689	15.1 [12.6–18.0]	1	** < 0.001**	1	**0.009**
Cohabiting	2092	44.1 [41.6–46.7]	1.33 [1.21–1.46]		1.14 [1.05–1.24]		1,856	37.4 [34.9–40.0]	2.24 [1.88–2.67]		1.32 [1.07–1.63]	
Level of education												
Less primary school	1238	53.0 [49.8–56.3]	1	** < 0.001**	1	0.25	647	33.9 [30.1–37.9]	1	**0.003**	1	**0.015**
Primary school	608	35.8 [31.7–40.3]	0.70 [0.63–0.77]		0.92 [0.82–1.02]		751	25.1 [22.0–28.5]	0.78 [0.67–0.91]		1.04 [0.90–1.20]	
Secondary school	856	31.7 [28.5–34.9]	0.60 [0.54–0.67]		0.91 [0.81–1.02]		852	28.2 [25.2–31.3]	0.85 [0.74–0.98]		1.11 [0.96–1.29]	
University studies	461	27.5 [23.4–32.1]	0.51 [0.44–0.60]		0.90 [0.76–1.06]		291	33.3 [28.0–39.2]	1.0 [0.83–1.21]		1.34 [1.11–1.61]	
Occupation												
Employed	503	31.9 [27.4–36.7]	1	** < 0.001**	1	0.21	1767	29.9 [27.6–32.3]	1	0.47	1	0.86
Unemployed	2663	40.5 [38.3–42.8]	1.34 [1.16–1.53]		1.09 [0.95–1.26]		776	27.6 [24.5–31.0]	1.04 [0.93–1.18]		1.01 [0.89–1.15]	
Residence												
Rural	1034	34.9 [31.5–38.5]	1	**0.004**	1	**0.023**	862	28.1 [24.8–31.7]	1	0.29	1	0.79
Urban	2138	41.2 [38.6–43.9]	1.18 [1.05–1.31]		1.13 [1.02–1.26]		1685	29.6 [27.2–32.2]	1.08 [0.93–1.23]		1.02 [0.89–1.16]	
Smoking												
Non-smoker	3150	39.1 [37.0–41.3]	1	0.12	1	0.07	968	28.1 [25.1–31.2]		** < 0.001**	1	0.15
Past smoker	9	49.7 [20.5–79.1]	1.34 [0.78–2.27]		1.23 [0.75–2.02]		813	37.1 [33.5–40.8]	1.25 [1.10–1.43]		1.05 [0.92–1.20]	
Current smoker	11	16.6 [4.9–43.4]	0.37 [0.12–1.16]		0.35 [0.13–0.96]		764	23.4 [20.3–26.8]	0.86 [0.74–1.02]		0.91 [0.78–1.05]	
Alcohol												
Non-user	3159	39.1 [37.0–41.3]	1	0.98	1	0.36	2091	29.4 [27.2–31.6]	1	0.62	1	0.54
User	10	35.9 [15.6–62.8]	1.01 [0.53–1.91]		1.26 [0.76–2.07]		454	28.1 [24.0–32.7]	0.96 [0.83–1.12]		0.96 [0.83–1.10]	
Fruits and vegetables												
Sufficient	734	38.4 [34.2–42.7]	1	0.97	1	0.76	610	30.6 [26.6–34.8]	1	0.31	1	0.42
Insufficient	2438	39.4 [37.0–41.7]	1.0 [0.90–1.12]		1.02 [0.92–1.12]		1937	28.7 [26.4–31.0]	0.93 [0.81–1.07]		0.95 [0.83–1.08]	
Sufficent activity												
Yes	1494	38.7 [35.8–41.6]	1	0.76	1	0.28	1845	26.6 [24.4–28.9]	1	** < 0.001**	1	**0.005**
No	1675	39.5 [36.9–42.1]	1.01 [0.93–1.10]		0.96 [0.89–1.03]		700	36.7 [33.1–40.5]	1.34 [1.20–1.49]		1.16 [1.05–1.30]	

Significant values are in bold.

*CI* confidence interval, *cPR* crude prevalence ratio, *aPR* adjusted prevalence ratio, *P* P-value, *MetS* metabolic syndrome.

## Discussion

To the best of our knowledge, this is the first nationwide study on MetS prevalence and MetS individual components in Algeria, according to the harmonized diagnostic criteria. Overall, MetS prevalence in Algeria was 34.0%. This prevalence was higher in women than in men and tended to be significantly associated with increasing age, cohabitation, women residing in urban areas, and men with higher levels of education and insufficient physical activity levels. Regardless of sex, abdominal obesity and low HDL-cholesterol were the most frequent metabolic disorders, whereas the clustering of low HDL-cholesterol, high blood pressure, and hyperglycaemia was the most prevalent MetS combination. Women were more likely to have abdominal obesity or low HDL-cholesterol levels, whereas men were more likely to have hypertriglyceridemia.

The estimated MetS prevalence was alarmingly high in Algeria, and there was comparable variation across the different definitions of MetS prevalence reported in the Eastern Mediterranean Region (EMR) and European populations. In the EMR, the estimated MetS prevalence according to the NCEP ATP III and IDF criteria was 32.9% and 34.6%, respectively, whereas the observed rates in Europe were 25.3% and 31.5%, respectively^6^. Our results were significantly higher than the pooled global MetS prevalence (12.5% [ATP III] and 28.2% [IDF])^6^, the prevalence in SSA (17.1% [ATP III] and 18.0% [IDF])^9^, and the prevalence in rural Algeria (17.4% [ATP III] and 25.7% [IDF])^14^. This increase in prevalence is due to the ongoing epidemiological transition occurring with rapid economic growth, wherein unhealthy lifestyles such as physical inactivity and unhealthy diets are increasingly common as part of the ongoing cultural globalisation^23^. Other factors specific to the Algerian population could account for the variation in MetS prevalence.

Our data showed a higher MetS prevalence in women than that in men. This could be a result of the high prevalence of abdominal obesity and low HDL-cholesterol levels (Fig. 2). In fact, central obesity and low HDL cholesterol, which are key predictors of MetS, have been shown to be generally higher in African women than in African men^24,25^. Other potential explanations for the higher MetS prevalence in women could be due to a longer life expectancy compared to men^26^ and because women are more prone than men to developing MetS in response to low socioeconomic status and work stress^27^. Moreover, recent observations indicate that men respond better than women to non-pharmaceutical strategies involving lifestyle measures and weight loss aimed at reducing MetS prevalence^28,29^. Comparable research in SSA^9^, African–American populations^30^, and Chinese populations^31^ showed a higher MetS prevalence in women than in men, which is consistent with our findings. In contrast, MetS prevalence was higher in men than in women in the general European population^32,33^. The alarmingly high MetS prevalence in both men and women in our study mirrors the limited progress toward achieving diet-related NCD targets in Algeria, where 38.6% of adult women and 23.3% of adult men live with obesity^25^.

As expected, MetS prevalence increased with increasing age, regardless of sex. Individuals aged 45 years and older were three times more likely to have MetS than younger adults aged 18–29 years. This clustering pattern suggests that MetS in young people may differ from that in older individuals, with differing prognostic and treatment implications given that many individuals develop metabolic risk factors by the time they are 65 years or older^34^. Our results were similar to those of studies that demonstrated that the effect of clustering MetS components increased with age^31,35–37^. In contrast, some studies have reported a levelling off in the rise of MetS prevalence with age, possibly due to a survivor effect whereby there is a relatively earlier death of young participants with MetS, causing a fall in prevalence in old age^38^.

Our analysis showed a positive association between MetS and cohabiting individuals, women in urban areas, men with higher levels of education, and men with low levels of physical activity. No association was found between occupation and insufficient fruit or vegetable intake. Socioeconomic disparities between men and women have been shown to be associated with different MetS prevalence rates. For instance, our findings are similar to those of the adult population of Saudi Arabia, where MetS had a positive association with income and higher education in men and unemployment in women^27^. In contrast, among Korean adults, men with the lowest education level, manual labourers, and those who were economically inactive were more likely to have MetS^39^. The higher likelihood of developing MetS in urban women in Algeria is probably a result of increased energy intake and reduced physical activity associated with urbanisation and cultural globalisation, which contribute to high obesity rates^40^. A higher MetS prevalence in urban areas has also been reported in SSA^9^, India^41^, and China^42^. In contrast, a study in the US reported a higher MetS prevalence in rural women^43^. This is possibly because the ongoing epidemiological transition has been experienced for longer in the US than in Algeria. Thus, it could be postulated that urban and rural differences in MetS in Algeria may disappear, and major efforts should not be made to reverse the disastrous lifestyle changes which have already occurred in urban areas.

The impact of dietary habits on MetS prevalence has been explored as a sex-related factor. For instance, Chinese men who had higher odds of having MetS had a high intake of meat and fried dough, while women at higher risk had higher salt and energy dietary patterns^44^. Although the close correlation between an unhealthy diet and increased triglyceride levels is well known, our study found no sex-related association between MetS and inadequate fruit and vegetable consumption after adjusting for the confounding factors. Whether the increased MetS prevalence in Algeria is due to specific dietary patterns warrants further investigation.

Taken together, our results suggest that MetS is very common in Algeria and that more attention needs to be paid to the sex-specific differences associated with MetS prevalence. Several population-based strategies aimed at reducing overweight, salt intake, and diabetes prevalence have been implemented in Algeria, but a multisectoral comprehensive nutrition plan is still lacking^45^.

## Strengths and limitations

This study is the first to present MetS prevalence and its individual components in the Algerian general adult population and provide insights into sex-specific factors associated with MetS using a nationally representative sample. However, our analysis had some limitations that warrant consideration. The cross-sectional design and assessment of some variables by self-reporting (physical activity, smoking, and diet metrics) could have led to misclassification errors, which may lead to underestimation of the true MetS prevalence. Smoking may have been under-reported because of the tendency to provide socially desirable responses. Our findings may provide evidence for sex- and age-specific initiatives to sustain and further improve the burden of MetS in Algeria.

## Conclusion

Overall, MetS prevalence and the prevalence of key MetS components, including abdominal obesity, low HDL cholesterol, and high blood pressure, is very high in adults with Algeria, especially women. To obtain maximal health gains and stem the CVD epidemic in Algeria, strategies targeting the reduction of MetS that focus on body mass and physical inactivity should be reinforced, particularly in urban women and men. There is a need for an increased multisectoral comprehensive nutrition plan as well as health promotion and prevention strategies targeting key determinants of MetS.

### Supplementary Information

Supplementary Information 1.

Supplementary Information 2.

## Data Availability

The database generated and used for analyses was provided by the WHO NCD Surveillance Monitoring and Reporting Unit. This dataset (Algeria.dta) and the do.file (Algeria.do) of the analysis report are available within the article (and its Supplementary Information files).
